# Efficacy and Safety of Eculizumab in Enteroaggregative *E. coli* Associated Hemolytic Uremic Syndrome

**DOI:** 10.3390/pediatric16010003

**Published:** 2024-01-04

**Authors:** Ratna Acharya, William L. Clapp, Kiran Upadhyay

**Affiliations:** 1 Division of Pediatric Emergency Medicine, Department of Pediatrics, Nemours Children’s Hospital, Orlando, FL 32827, USA; ratna.acharya@nemours.org; 2 Division of Anatomic Pathology, Department of Pathology, University of Florida, Gainesville, FL 32610, USA; clapp@pathology.ufl.edu; 3 Division of Pediatric Nephrology, Department of Pediatrics, University of Florida, Gainesville, FL 32610, USA

**Keywords:** hemolytic uremic syndrome, eculizumab, enteroaggregative, *E. coli*, children

## Abstract

Background. Hemolytic uremic syndrome (HUS) may present atypically without the full triad of classical HUS. Eculizumab has been shown to be efficacious in complement-mediated atypical HUS and some cases of Shiga-toxin (ST) associated HUS. We report the utility of eculizumab in enteroaggregative *E. coli* (EAEC) associated HUS. Case summary. A female toddler presented with hemolytic anemia, oliguric acute kidney injury (AKI) without thrombocytopenia, and peripheral schistocytes. The stool examination for ST was negative but positive for EAEC. She required several hemodialysis sessions and received one dosage of eculizumab with rapid reversal of AKI and hemolytic markers. A kidney biopsy revealed acute tubular injury and segmental glomerular basement membrane splitting. Genetic testing was negative for complement mutations or deficiencies. A follow-up six months later showed persistently normal renal function and hematological markers. Conclusion. The clinical and histological manifestations of non-ST-associated diarrheal HUS and the role of eculizumab in this condition warrant future larger studies.

## 1. Introduction

Historically, the term hemolytic uremic syndrome (HUS) refers to a triad of acute kidney injury (AKI), thrombocytopenia, and microangiopathic hemolytic anemia (MAHA) [1]. Recently, a few reports of HUS without the full triad, also called partial HUS, have been reported [2].

The 2011 German outbreak of Shiga-toxin (ST) producing *E. coli* (STEC) associated HUS was caused by serotype 0104:H4 which shared the genetic elements with enterohemorrhagic and enteroaggregative *E. coli* (EAEC) [3]. EAEC comprises a variety of serotypes that possess enterotoxins, hemolysins, fimbriae, and outer membrane proteins involved in the adhesion process [4]. EAEC can cause acute and persistent watery diarrhea leading to outbreaks but can also be isolated from the stool of healthy children [5]. 

Complement dysregulation and/or overactivation either due to antibodies to various complement factors or gene variants of various complement proteins have been well described in atypical HUS (1). The efficacy and safety of eculizumab in patients with atypical HUS is well known [6]. Eculizumab is a humanized monoclonal antibody that prevents C5 cleavage and the formation of C5a and C5b-9, thus preventing overactivation of the complement system. In addition to its benefits in complement-mediated atypical HUS, its efficacy has also been described in children with ST-HUS, mainly in those with central nervous system (CNS) or cardiac involvement [7,8]. Recently, complement inhibitors such as eculizumab and ravulizumab have also been shown to be efficacious in thrombotic microangiopathy (TMA) associated with the coronavirus disease of 2019 (COVID-19) [9,10]. Also, in children previously treated with eculizumab for atypical HUS, switching to ravulizumab has been shown to result in stable kidney and hematologic parameters, without any safety concerns [11]. 

We report a toddler with oliguric AKI, hemolytic anemia without thrombocytopenia, and peripheral schistocytes, in the absence of thrombotic features on renal biopsy in association with non-ST-producing EAEC with an excellent response to eculizumab. 

## 2. Case Presentation

A previously healthy female toddler presented with several episodes of emesis and decreased urine output. There was no history of diarrhea or bloody stool, intake of contaminated food or water, or international travel. She was born prematurely at 30 weeks and was fully immunized. Past medical history was unremarkable for solid organ or bone marrow transplantation, malignancy, autoimmune disorders, intake of nephrotoxic drugs, infections, or hypertension. There was no family history of renal disease. Prior renal function showed a serum creatinine of 0.3 mg/dL at one month of life. 

Vital signs showed an afebrile child with a pulse of 140 beats/minute and blood pressure of 88/56 mm Hg (<50th percentile). Physical examination was unremarkable except for signs of mild dehydration. 

**Figure 1:**
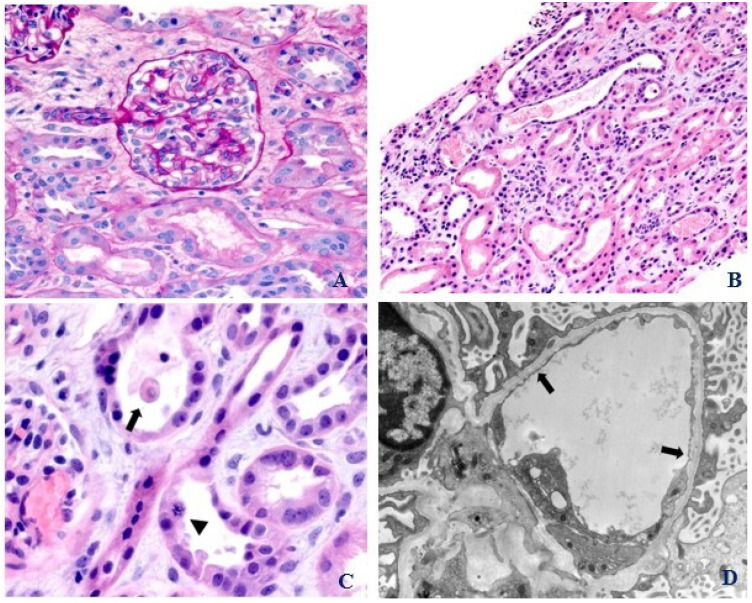
(**A**) Renal biopsy. Light microscopy. Normal appearing glomerulus and hilar arteriole (PAS, 400×). (**B**) Renal biopsy. Light microscopy. Acute tubular injury with tubular dilatation and epithelial attenuation (H&E, 200×). (**C**) Renal biopsy. Light microscopy. Acute tubular injury: epithelial cell sloughing (arrow) and mitotic figure (arrowhead) (H&E, 630×). (**D**) Renal biopsy. Electron microscopy. Glomerular capillary loop with glomerular basement membrane splitting (arrows) (EM, 12,000×).

Initial investigations upon presentation are shown in Table 1. Due to progressive decline in renal function, a kidney biopsy was performed on day two of admission which showed 39 normocellular glomeruli with no segmental sclerosis, necrosis, crescent, mesangiolysis, or capillary thrombosis (Figure 1A). There were patchy areas of acute tubular injury (ATI) characterized by tubular dilation, epithelial attenuation, mitotic figures, and sloughing of cells (Figure 1B,C). There were no tubular necrosis and significant cellular infiltrates. Immunohistochemical stains for myoglobin and hemoglobin were non-contributory. Immunofluorescence was negative for immunoglobulins and complements. Electron microscopy showed intact podocyte foot processes, the absence of electron-dense deposits, and segmental glomerular basement membrane (GBM) splitting without multi-lamination (Figure 1D). There were no platelet or fibrin thrombi of the microvasculature, vascular mucoid intimal edema, onion skinning, detachment of glomerular endothelial cells, and “double contours”. The peripheral blood flow cytometry showed normocytic anemia with no clonal B cells, abnormal T cells, or blasts. Bone marrow biopsy showed normocellular bone marrow (90–95%) with no increase in blasts. 

On day three of the presentation, bloody stools appeared which led to a high index of suspicion for HUS. Stool examination was negative for ST but positive for EAEC (*BioFire FilmArray GI PCR panel*). We could not specify the serotyping as it was not available commercially. Serum complements C3 and C4 were normal. A genetic test for atypical HUS was sent for gene variants of various complement proteins. ADAMTS13 activity was normal. The platelet counts remained normal, and the peripheral blood smear continued to show the absence of schistocytes. 

The differential diagnoses included partial HUS, and AKI secondary to severe volume depletion induced by vomiting, non-microangiopathic hemolytic anemias, and malignancies. Although AKI and histological features of tubular injury could be due to severe volume depletion from vomiting, the presence of concurrent hemolytic anemia warranted a high index of suspicion for conditions such as partial HUS. 

**Figure 2:**
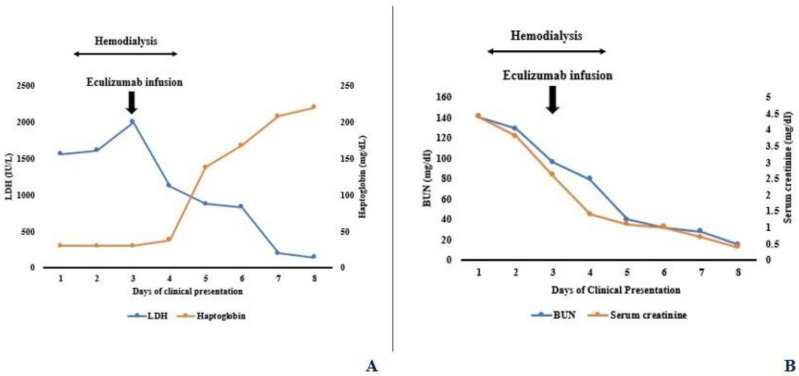
(**A**) Rapid normalization of hematological markers after administration of eculizumab. (**B**) Rapid normalization of renal function after administration of eculizumab.

Isotonic intravenous (IV) saline with bicarbonate was initiated upon admission. However, the renal function continued to worsen with peak serum creatinine 4.4 mg/dL and BUN 141 mg/dL. Given oligoanuria and electrolyte abnormalities, she received three sessions of daily hemodialysis (HD) with unfractionated heparin for anticoagulation. Loop diuretic was not administered given concern for dehydration-induced oliguria. On day three of admission, a single dose of IV eculizumab 300 mg was given after administration of meningococcal vaccine and penicillin therapy, with rapid recovery of renal function and hemolytic markers (Figure 2A,B). No further dosages of eculizumab were required. Blood pressure remained normal. A follow-up of laboratory testing showed the absence of autoantibodies to factor H and I, normal serum factor H and I levels, and elevated soluble complement 5B-9 (SC5B-9) levels. Atypical HUS genetic susceptibility panel showed no deletion or duplication of *C3*, *C4BPA*, *CD46*, *CD59*, *CFB*, *CFH*, *CFI*, *CFHR5*, *DGKE*, *THBD*, *PLG,* and *MMACHC* genes (Cincinnati Children’s Clinical Laboratories, Cincinnati, OH, USA). Platelet counts did not decrease throughout the hospital course. Urinalysis continued to show dipstick positive for blood but absence of microscopic hematuria along with 1+ to 2+ proteinuria. Urine beta-2 microglobulin was elevated (3200 mcg/L, normal: <300 mcg/L, Mayo Clinic Laboratories, Rochester, MN, USA). Serum albumin remained in 3.5–4 g/dL range. Serum uric acid returned to normal following hemodialysis and stayed normal with no need for anti-hyperuricemic therapies such as rasburicase. 

In a short-term follow-up of six months, the patient was normotensive with normal renal function and blood counts with resolution of tubular proteinuria. There were no medication-related adverse events. Follow-up genetic testing showed no complement or factor deficiencies and the absence of complement gene mutations. 

## 3. Discussion

Classic thrombotic microangiopathy (TMA) presents with non-immune microangiopathic hemolytic anemia, thrombocytopenia, and organ dysfunction [1]. The two major syndromes that fall under the spectrum of TMA are HUS and thrombotic thrombocytopenic purpura (TTP), based upon organ dysfunction; kidneys in HUS and brain in TTP. However, it is important to understand that not all cases of TMA present all three features. A minimum of three of the following features are needed to define TMA: anemia, elevated LDH, decreased haptoglobin, peripheral schistocytes, and thrombocytopenia [12]. Some studies have coined the term “partial” HUS for those presenting without thrombocytopenia and/or schistocytes [13,14]. A retrospective review of 128 TMA patients showed a 55% and 43% incidence of thrombocytopenia and peripheral schistocytes respectively [14]. Hence, thrombocytopenia and peripheral schistocytes may not be present in all cases of TMA. Table 2 shows the synopsis of classic and “partial” HUS. Balestracci et al. reported a 5.6% incidence of absence of thrombocytopenia in patients with post-diarrheal HUS [15]. Sellier-Leclerc et al. reported that 15% of atypical HUS patients with complement mutation had normal platelet counts [16]. In one study of HUS patients associated with anti-factor H autoantibody, toxins, cancer, graft, autoimmune, ST, and other infections, the incidence of thrombocytopenia was 13%; children were not included in this study [2]. LDH level and schistocyte counts were higher in the thrombocytopenic group. There was no difference in serum creatinine, hemoglobin, and proteinuria between the thrombocytopenic and non-thrombocytopenic groups. Both groups were treated with plasmapheresis and there was no difference between the number of plasmapheresis sessions between the groups. Renal outcome was similar in both groups [2]. Similarly, the absence of peripheral schistocytes in patients with biopsy-proven TMA has been described [17]. 

Renal-limited TMA is manifested by distinct thrombi and histological changes of TMA on biopsy without other findings such as MAHA or thrombocytopenia [18]. This has also been described in oncology patients who are treated with anti-vascular endothelial growth factor (VEGF) therapies. The possible mechanisms of renal-limited TMA in these patients are direct VEGFA inhibition by antibody binding or VEGF trap (a soluble decoy receptor) or via inhibition of the mammalian target of the rapamycin pathway [19]. Histologically, classic renal-limited TMA is characterized by intimal proliferation and/or endothelial swelling with luminal fibrin deposition in the vessels. Mesangiolysis, GBM duplication, red-cell fragments, double contours of the capillary basement, arterial thrombosis, mucoid intimal edema, and vascular onion skinning are typically found in renal TMA [14]. Electron microscopy may show glomerular capillary subendothelial expansion with a new basement membrane formation [18]. However, the immunofluorescence findings are predominantly negative in TMA as complement activation occurs on the cell surfaces as opposed to a large-scale fluid-phase activation in membranoproliferative glomerulonephritis [20,21]. In one study of TMA patients, renal biopsy showed glomerular-only TMA in 38%, renal vascular TMA in 62% and both glomerular and vascular TMA in 51% [14]. Focal and diffuse ATI were seen in 47% and 28% respectively [14]. In those with absent schistocytes, ATI was present in 70% and GBM duplication in 76% [14]. Our patient met the laboratory criteria for TMA but did not have the typical histologic findings of renal TMA except for ATI and GBM splitting. It needs to be studied whether the latter features are the under-recognized histological manifestations of renal TMA and may warrant inclusion in any future reclassification of TMA. Indeed, the absence of renal thrombi in some cases of renal TMAs has been reported, and reclassifying renal TMA to microangiopathy with or without thrombosis has been proposed [22]. There may also be a correlation between the peripheral schistocytes and the renal histology in patients with TMA. In one study, the presence of peripheral schistocytes was strongly associated with the typical histological findings of acute TMA [14]. Segmental GBM splitting, as seen in our patient, has not been described and its exact pathogenesis is unknown. 

The efficacy of eculizumab in diarrhea-associated HUS has been reported. Lapeyraque et al. described 3 children with severe STEC-HUS who were treated with eculizumab due to CNS involvement [7]. There was a rapid normalization of platelet counts and LDH levels and all of them had renal and CNS recovery. Alternative complement pathway activation leading to the inflammatory and prothrombotic cascade has been shown to occur in ST-HUS [23]. Also, ST interacts with complement regulators of the factor H protein family providing a rationale for using eculizumab [24]. With regards to the usage of eculizumab in EAEC colitis, not much data is available. A retrospective registry analysis showed no beneficial effects of eculizumab as compared to plasmapheresis alone in the treatment of the 2011 German outbreak of EAEC HUS [3]. In contrast, the efficacy of eculizumab was demonstrated in 9 patients with STEC 0104:H4, 0–4 days after HUS diagnosis [25]. Also, the exact mechanism of EAEC-associated AKI and hemolytic anemia is not known. They either inactivate the complement cascade enhancing their survival or activate the complements leading to tissue damage [26]. Despite complement activation, various complement evasion strategies may be utilized by organisms such as having a capsule that prevents complement-mediated bacteriolysis, secretion of proteases that inactivate the complements, and acquisition of host regulatory complement proteins that downregulate the complement pathways [26]. Indeed, EAEC produces a serine protease called Pic, which mediates immune evasion by the direct cleavage of complement [26]. 

In cases of hemolytic anemia, it is sometimes difficult to differentiate extravascular hemolysis vs. intravascular hemolysis as both will present with anemia, elevated serum LDH, increased reticulocyte count, and increased serum unconjugated (indirect) bilirubin. The presence of splenomegaly and examination of peripheral smear showing spherocytes are most commonly seen with extravascular hemolysis. Direct antiglobulin test for C3 and IgG helps in identifying various causes of autoimmune hemolytic anemia and may indicate extravascular hemolysis; this test was negative in our patient. Intravascular hemolysis is more favored in those with undetected plasma haptoglobin, the presence of elevated free plasma hemoglobin, and the absence of hematuria (>5 red blood cells/hpf) but positive dipstick for blood and/or the presence of urinary hemosiderin. The patient described in this report most likely had intravascular hemolytic anemia given the presence of the finding’s characteristic of intravascular hemolysis. Complement-mediated hemolysis is seen with paraoxysmal nocturnal hemoglobinuria (PNH), cold agglutinin disease, paraoxysmal cold hemoglobinuria, and some types of warm antibody autoimmune hemolytic anemias [27]. PNH is an important rare cause of complement-induced intravascular hemolytic anemia and may present with thrombotic manifestations; renal injury is unlikely although rare cases of concurrent renal injury have been described [28]. Hemosiderin deposition in the renal tubular cells is the most consistent histological finding in patients with PNH and renal injury [28]. PNH is due to the deficiency of glycosyl phosphatidylinositol (GPI)-anchored complement regulatory proteins such as CD55 and CD59. CD55 exerts an inhibitory function at the C4–C3 level in the classic and alternate complement pathway, and CD59 blocks the assembly of the C5b–C9 complex at the C8–C9 stage [29]. The Ham’s and sucrose lysis tests along with flow cytometric evaluation for CD55 and CD59 help confirm the diagnosis. The patient described in this report had negative immunohistochemical staining for hemoglobin in the kidney biopsy specimen and had normal flow cytometry. The Ham’s and sucrose lysis tests were not performed.

The limitations of our report include the absence of serotyping data and short-term follow-up. Also, one would speculate that the patient might have sustained transient AKI and hence could have had spontaneous clinical improvement even without the usage of eculizumab. However, given the presence of concurrent non-immune hemolytic anemia and elevated SC5B-9, the authors believed that this patient had a pathology leading to complement activation, and hence the complement inhibitor was used. Given the absence of classic presentation of renal histology seen in TMA affecting kidneys, future studies will need to examine these possibly under-recognized histological manifestations of TMA. What we can infer from this report is that eculizumab seems to expedite the recovery of renal function and hematological markers without any adverse events in EAEC-associated “partial” HUS. 

## 4. Conclusions

A high index of suspicion for “partial” HUS is necessary for normotensive patients with otherwise unexplained non-microangiopathic hemolytic anemia with AKI. Our report is also novel given that vomiting was the presenting symptom; diarrhea appeared later after the onset of AKI and hemolytic anemia. Although EAEC can also be isolated from healthy children, the association of other clinical and laboratory features in this report may point towards the potential cause-and-effect relationship between EAEC and HUS. The pathophysiology, clinical course, prognosis, and efficacy of complement inhibitors in non-ST-producing EAEC-associated “partial” HUS need to be studied further in larger studies. 

## Figures and Tables

**Table 1:** Table showing initial investigations upon presentation.

Investigations	Values	Normal Values
WBC count	17.3 × 10^9^/L	4–11 × 10^9^/L
Hemoglobin	6.3 g/dL	10.5–13.5 g/dL
Platelet count	343 × 10^9^/L	150–450 × 10^9^/L
LDH	1565 IU/L	135–225 IU/L
Haptoglobin	<30 mg/dL	40–215 mg/dL
Peripheral smear	Absence of schistocytes, spherocytes	
Direct antiglobulin test, C3 and IgG	Negative	
ADAMTS13 activity	80%	>60%
C3 complement	95 mg/dL	87–200 mg/dL
C4 complement	21 mg/dL	13–50 mg/dL
SC5B-9 level	469 ng/mL	≤244 ng/mL
Genetic testing for atypical HUS	Negative for complement mutations or deficiencies	
Uric acid	12 mg/dL	2.6–6.8 mg/dL
Serum sodium	134 mmol/L	135–145 mmol/L
Serum potassium	5.4 mmol/L	3.5–4.5 mmol/L
Serum bicarbonate	12 mmol/L	22–30 mmol/L
BUN	114 mg/dL	6–21 mg/dL
Serum creatinine	3.1 mg/dL	0.20–0.43 mg/dL
Serum calcium	8.7 mg/dL	8.4–10.2 mg/dL
Serum phosphorus	7/8 mg/dL	4.3–6.8 mg/dL
Serum albumin	3.2 g/dL	3.5–5.2 g/dL
ALT, AST	23 IU/L, 118 IU/L	0–35 IU/L, 0–37 IU/L
Total serum bilirubin	1.8 mg/dL	0–1 mg/dL
Sickle cell screen	Negative	
Serum folate and vitamin B12 levels	Normal	
G6PD deficiency	Absent	
Respiratory virus panel, including SARS-CoV-2 PCR	Negative	
EBV DNA PCR, CMV DNA PCR	Negative	
PTH	105 pg/mL	12–88 pg/mL
Iron saturation	41%	20–55%
Stool test	Positive for EAEC, negative for Shiga-toxin	
Urinalysis	2 RBC/hpf, 2 WBC/hpf, trace proteinuria, negative nitrites and leukocytes, pH 7, specific gravity 1.025	
Plasma free hemoglobin	190 mg/dL	<150 mg/dL
CRP	44 mg/L	0–5 mg/L
Procalcitonin	36.37 ng/mL	<0.1 ng/mL
Cultures, blood, and urine	Negative	
CK	161 U/L	0–180 U/L
ANA, ANCA, Anti-GBM antibody	Negative	
Renal bladder sonogram	Enlarged, echogenic kidneys measuring 9–9.5 cm in length, normal Doppler examination with no evidence of renal vein thrombosis	
Abdominal sonogram	No splenomegaly or hepatomegaly	

WBC: White blood cell; LDH: lactate dehydrogenase; ADAMTS13: a disintegrin-like metalloproteinase with thrombospondin motif type 1; SC5B-9: soluble complement 5b-9; HUS; hemolytic uremic syndrome; BUN: blood urea nitrogen; ALT: alanine aminotransferase; AST: aspartate aminotransferase; G6PD: glu-cose-6 phosphate dehydrogenase; SARS-CoV-2: severe acute respiratory syndrome coronavirus 2; EBV: Epstein-Barr virus; CMV: cytomegalovirus; PCR: polymerase chain reaction; PTH: parathyroid hormone; EAEC: enteroaggregative *E. coli*; RBC; red blood cells; CRP: C-reactive protein; CK; creatine kinase; ANA: antinuclear antibody; ANCA; anti-neutrophil cytoplasmic antibody; GBM: glomerular basement membrane.

**Table 2:** A synoptic table comparing elements of classic HUS, partial HUS, and the index case.

	Classic HUS	Partial HUS	Index Case
Anemia	Present	Present	Present
Thrombocytopenia	Present	May be absent	Absent
Peripheral schistocytes	Present	May be absent	Absent
LDH	Elevated	Elevated	Elevated
Haptoglobin	Low	Low	Low/Undetected
Acute Kidney Injury	Present	Present	Present
Renal pathologic lesions of classic TMA	Present	May be absent	Absent

## Data Availability

Data is contained within the article.
